# Gametogenesis-Related Fluctuations in Ovothiol Levels in the Mantle of Mussels from Different Estuaries: Fighting Oxidative Stress for Spawning in Polluted Waters

**DOI:** 10.3390/biom10030373

**Published:** 2020-02-28

**Authors:** Oihane Diaz de Cerio, Lander Reina, Valeria Squatrito, Nestor Etxebarria, Belen Gonzalez-Gaya, Ibon Cancio

**Affiliations:** 1 CBET Research Group, Department of Zoology & Animal Cell Biology, Faculty of Science & Technology (FCT-ZTF) and Research Centre for Experimental Marine Biology & Biotechnology (PiE-UPV/EHU), University of the Basque Country (UPV/EHU), EMBRC-Spain, Areatza Hiribidea 47, 48620 Plentzia, Basque Country, Spain; oihane.diazdecerio@ehu.eus (O.D.d.C.); lanreiher@gmail.com (L.R.); valeria.squatrito@studio.unibo.it (V.S.); 2 IBEA Research Group, Department of Analytical Chemistry, Faculty of Science & Technology (FCT-ZTF) and Research Centre for Experimental Marine Biology & Biotechnology (PiE-UPV/EHU, University of the Basque Country (UPV/EHU), EMBRC-Spain, Areatza Hiribidea 47, 48620 Plentzia, Basque Country, Spain; nestor.etxebarria@ehu.eus (N.E.); belen.gonzalez@ehu.eus (B.G.-G.)

**Keywords:** *Mytilus galloprovincialis*, ovothiol, ovothiol synthase, glutathione, oxidative stress, metabolome, oocytes, pollution biomonitoring

## Abstract

Reactive oxygen species present a challenge for marine organisms releasing gametes into the water. Thiol-containing molecules protect cells against oxidative stress, and ovothiol (OSH), an antioxidant-reducing mercaptohistidine, has been described as especially relevant in the oocytes of marine invertebrates. Ovothiol synthase (*ovoA*), in charge of the first step in OSH synthesis, was sequenced in mussels, *Mytilus galloprovincialis*. Transcription levels of *ovoA* in mantle did not significantly change along the reproductive cycle. No alterations of *ovoA* transcription were observed after a laboratory copper (10 µg/L) exposure or in mussels captured in a highly polluted site. Conversely, the metabolomic analysis of the hydrophilic metabolite content in mantle clearly classified mussels according to their site of origin, especially at the most advanced stages of oogenesis. Quantification of OSH-A and -B and glutathione (GSH), revealed stable levels in mantle at early gametogenesis in the unpolluted sampling site, but a strong increase in female mantle previous to spawning in the polluted site. These increased concentrations under pollution suggest that OSH-A accumulates along oogenesis, independent of gene transcription regulation. The concerted accumulation of OSH-A and GSH suggests the building of a balanced cellular redox-system to scavenge ROS produced in the oocyte before and during fertilization.

## 1. Introduction

The little we know about ovothiol (OSH) or π-*N*-methyl-5-thiohistidine alludes to a potential “wonder molecule” [1,2]. Although it was discovered in the 1980s [2,3], its history needs to be traced back to 1930 when Otto Warburg, as a visiting scientist in Stazione Zoologica Anton Dohrn in Naples, before his Nobel Prize award in Medicine in 1931, described the oxidative burst occurring in sea urchin oocytes upon fertilization. Sperm fusion in sea urchins results in an explosive production of extracellular H_2_O_2_ for the generation of the fertilization envelope [4]. An accompanying increase in the intracellular concentration of H_2_O_2_ needs to be counteracted by antioxidant mechanisms. It was also in Naples that 50 years later, OSH would be discovered in the oocytes of the sea urchin *Paracentrotus lividus*, hence the name [2,3]. OSH was proposed to have a physiological role in protecting the urchin *Strongylocentrotus purpuratus* eggs from H_2_O_2_ toxicity [5,6]. OSH would function as a non-enzymatic scavenger of H_2_O_2_, the resulting oxidized OSH disulphide being reduced back by glutathione (GSH) [2]. OSH has since been described in ovaries, oocytes and biological fluids of different marine invertebrates, but also in proteobacteria, cyanobacteria and in some fungi and protists, including protozoa and algae [1,5,6,7,8,9]. Three different isoforms have been described, depending on their level of methylation: ovothiol A (OSH-A) and ovothiols B and C (OSH-B and -C), which are the mono- and di-methylated forms of OSH-A, respectively [2,9].

OSHs exhibit quite special chemical properties because of the reactivity of the functional thiol, in relation to its positioning into the imidazole system, conferring stronger reactive oxygen species (ROS) scavenging activity compared to other thiols, with reactivity also against hydroperoxides and peroxynitrites [2]. This thiol group, characterized by a remarkable acidity, is attached to position 5 of the imidazole ring, providing a pK_a_ much lower (pK_a_ = 1.4) than cysteine (pK_a_ = 8.4), GSH (pK_a_ = 8.7), trypanothione (pK_a_ = 7.4) and coenzyme A (pK_a_ = 9.8). This unique property has led to the belief that OSH-A may be a particularly efficient peroxide scavenger [1,2,5,6]. On the other hand, the disulphide of OSH-A is less stable than the disulphide of GSH. Unlike most thiols, OSHs are rapidly oxidized by H_2_O_2_, in a reaction at least twice as fast as that with GSH. The oxidation is followed by a reaction with another molecule of reduced OSH to form a disulphide [2]. Therefore, it seems that the more reactive OSH-A and the more reductive GSH could cooperate to protect the organism from H_2_O_2_-induced damage [1,2,6]. Due to its chemical properties as a ROS scavenger and its potential therapeutic applications in humans, the interest in OSH-A has grown lately. For example, OSH-A was shown to reduce cell proliferation and increase autophagy in the Hep-G2 human liver carcinoma cell line [10]. In the same way, it has been shown to inhibit collagen deposition and reduce liver fibrosis, thus displaying a hepatoprotective capacity [11].

OSH-A has been shown to have a role during development, protecting sea urchins from environmental factors along early development in seawater [9]. In the pathogenic protozoa *Leishmania donovani* and in the non-human infective protist *Crithidia fasciculate*, OSH-A has been suggested to participate in the protection of parasites from oxidative stress produced by macrophages during infection [8]. The unmethylated histidine derivative of OSH-A, 5-thiolhistidine, was reported as part of the mixture that forms adrenochrome, the iron (III)-containing pigment in the branchial heart of *Octopus vulgaris* [7]. Moreover, the metabolite L-ovothiol A disulphide could act as a male pheromone during mating in the marine polychaete *Platynereis dumerilii* [12], and as a redox regulator in chloroplasts in the microalga *Dunaliella salina,* where OSH-A disulphide inactivates ATPase by a disulphide exchange reaction [13].

The first step of the OSH-A biosynthetic pathway involves the conjugation of cysteine and histidine accompanied by the net transfer of the cysteine sulfur atom into the C_5_–H bond in the histidine side chain, resulting in the formation of a 5-L-histidyl-L-cysteine sulfoxide conjugate [14]. The aforementioned reaction is catalyzed by an iron (II)-dependent sulfoxide synthase, termed A synthase (OvoA). The second cleavage step is catalyzed by a lyase (OvoB) that generates a thiohistidine. Then, OvoA again catalyzes the third step, methylating the imidazole ring to produce OSH-A [1,2,9,14].

Recent studies have reported the characterization of *ovoA* gene sequence and OvoA protein primary structure in several species of marine invertebrates [9,15]. Similar to ergothioneine synthase (EgtB), the other known sulfoxide synthase that contributes to the biosynthesis of the biothiol compound ergothioneine, OvoA shows a conserved N-terminal DNA damage-inducible (DinB) superfamily domain characterized by a four-helix bundle at the N-terminal region. This domain contains a putative iron-binding motif (HX3HXE) and is involved in the SOS response [9]. The DinB domain is followed by a domain structurally related to that of copper-dependent formyl glycine-generating enzymes (FGE-sulfatase), which performs the conversion of cysteine residues. Unlike EgtB, OvoA contains an additional S-adenosylmethionine methyltransferase (SAM-transferase) domain at the C-terminal region [9,15]. This domain is related with various functions such as transmethylation and trans-sulfuration. It could also take part in the methylation of OSH to generate its different forms [2,9,15]. *ovoA* is highly conserved in marine metazoans, both in protostomes and in deuterostomes [9,15]. There have been independent events that resulted in the loss of the *ovoA* gene in various groups, notably in nematodes and arthropods, and in the ancestor of teleost fish and tetrapods [9,15]. In Hydrozoa and Rotifera Bdelloidea, the gene has been obtained through lateral gene transfer after previous gene reduction events [15].

The promoter region of the sea urchin *ovoA* gene contains metal, stress and oxyradical response elements that regulate its transcription under stress conditions [9]. It is this capacity to respond to environmental variables which confers metazoans’ adaptability under stress, and that constitute interesting study processes for environmental toxicologists. For instance, toxins and heavy metals dispersed in seawater can induce oxidative stress and the resulting ROS and the metals themselves may activate transcription factors to regulate *ovoA* transcription.

Marine mussels are exceptionally well adapted to live in coastal marine habitats, where they are exposed to fluctuating environmental conditions and elevated levels of natural and anthropogenic pollutants. For these reasons, they possess an antioxidant system that plays a key role in their remarkable capacity for acclimation [16,17,18]. In this study, the mussel (*Mytilus galloprovincialis*) was studied due to its relevant position as a pollution sentinel species in estuaries and coastal waters, where it is used in pollution monitoring programs. Mussels are filter-feeders, which results in the accumulation of high levels of contaminants in their tissues, sometimes well over concentrations found in the water column [17]. In health assessment, mussels are used to measure biological endpoints that could provide information about the level of exposure to pollutants, or the effects of such exposures [17,18,19,20]. In this sense, there are different programs using oxidative stress responses to evaluate toxicological effects of different anthropic activities, accidental spills [17,20,21] or the effects of climate change [22].

*ovoA* orthologs have been sequenced in different molluscs: the pacific oyster (*Cassostrea gigas*), the Californian sea hare (*Aplysia californica*), the yesso scallop (*Mizuhopecten yessoensis*) and the air-breathing freshwater snail (*Biomphalaria glabrata*) [15]. Consequently, the aim of the present study was to sequence the still unknown coding sequence of the *ovoA* gene in *Mytillus galloprovincialis* based on the previous data. Furthermore, the patterns of *ovoA* transcription and OSH-A accumulation were analyzed in the male and female mussels collected in two different estuaries with different pollution burdens, Plentzia and Arriluze, along a whole gametogenic cycle. In marine organisms, such as mussels, several metals interact with molecules containing sulfhydryl groups as OSH, the classical metal scavenger being metallothioneins [19]. In this context, we further studied the transcriptional regulation of *ovoA* under acute laboratory exposure to copper, a known pro-oxidant. This was completed with a non-target metabolomics study (Liquid Chromatography-High Resolution Mass Spectrometry) to distinguish mantle tissues along gametogenesis in different estuaries, together with a targeted analysis of OSHs and GSH in order to quantify their levels in those tissues.

## 2. Materials and Methods

### 2.1. Sampling of Mussels along Their Gametogenic Cycle

Thirty mussels (*Mytilus galloprovincialis*) were collected monthly at low tide from August 2017 to March 2018 in two localities of the Basque Country (Spain). One of the sampling points was in Arriluze (43°20′20.53″ N–3°0′46.15″ W), a putatively polluted site in the Nerbioi estuary, and the second one in Plentzia (43°24′32.35″ N–2°56′50.32″ W) in the Butroe estuary. Genetic material was collected and utilized according to Access and Benefit Sharing Legislation in place in Spain and under the Internationally Recognized Certificate of Compliance (ABSCH-IRCC-ES-248276-2). 

Mussels were dissected in the laboratory shortly after collection. The mantle within each valve was separated using one half for histological studies by fixation in 10% Neutral Buffered Formalin (10% NBF) for 24 h. The other half of the mantle was immediately frozen at −80 °C after embedding in RNAlater.

### 2.2. Exposure of Mussels to Copper under Laboratory Conditions

One hundred mussels (*M. galloprovincialis*) were collected at the end of April in Plentzia at the Butroe estuary (43°24′32.35″ N–2°56′50.32″ W). All the mussels were acclimatized for 1 week to laboratory conditions in continuously aerated seawater at 18 °C and a photoperiod of 12 h/12 h. The seawater was decanted, filtered through 0.2 µm filters and treated by UV light. After acclimatization, 50 mussels were taken as control organisms and placed in aquaria with clean seawater. A further 50 mussels were placed in 25 L aquaria and exposed to copper (II) oxide (CuO) at a final concentration of 10 μg/L. Samplings from control and Cu-treated mussels were performed at 3 and 7 days of exposure. At each time-point, mantle, digestive gland, gills and foot were dissected for molecular analyses from 25 mussels in the control and Cu exposure groups. Tissues were immediately frozen at −80 °C after embedding in RNAlater. Mantles were also collected for histological analysis and fixed in 10% NBF.

### 2.3. Histological Processing and Microscopic Analysis of Gonads

After 24 h fixation, mantle samples were transferred to 70% ethanol and then processed for paraffin embedding following an automated program in a Leica AS300S tissue processor. Paraffin blocks were cut at a thickness of 5 μm using a Leitz 1512 microtome (Leica, Vienna, Austria). Sections were then automatically stained with hematoxylin/eosin using a Leica AutostainerXL (Leica) to finally mount the slides with DPX (Sigma-Aldrich, St. Louis, MO, USA) for microscopic visualization. Sex and developmental stage of each animal was identified within any of the six gamete developmental stages (1 = resting, 6 = spawning) described by Seed [23]. Histological analysis revealed no lesions nor any meaningful infestation or disease.

### 2.4. Cloning of the Ovothiol Synthase Coding Domain Sequence

The sequence of Purple sea urchin (*Strongylocentrotus purpuratus*) *ovothiol synthase*, (XM_784225.4) was used to search for ortholog sequences in the GenBank database through BLASTn analysis. The hits corresponding to the species phylogenetically more related to mussels were selected for primer design: pacific oyster (*Crassostrea gigas*, JH818685), sea urchin (*Paracentrotus lividus*, KT900888), California sea-hare (*Aplysia californica*, XM_005099513), yesso scallop (*Mizuhopecten yessoensis*, XM_021501961) and the air-breathing freshwater snail (*Biomphalaria glabrata*, XM_013228453). Then, Clustal Omega was used for multiple sequence alignment and selection of homologous sequence fragments with the highest degree of sequence conservation across species. This information was used to design degenerate primers (FW 5′-3′ ATGTTTGARACDGGWGTKGATGAAATG and RV 5′-3′ CCATAYTCATTGTCCADCC) using Eurofins genomics.

### 2.5. PCR and Electrophoresis

The amplification was performed using a commercial Taq DNA Polymerase recombinant Kit (Invitrogen, Thermo Fisher Scientific) for 35 cycles in a 2720 Thermal Cycler (Applied Biosystems, Carlsbad, CA, USA), using, as a template, cDNA coming for a pool of mussel ovaries that was previously synthesized in the framework of another project and stored at −80 °C. The thermocycler was programmed with the following steps: 94 °C for 2 min, denaturation at 94 °C for 30 s, annealing at 50 °C (Tm) for 30 s, elongation at 72 °C for 8 s and finally, 72 °C for 8 min. PCR products were analyzed by electrophoresis in ethidium bromide-stained agarose gels (1.5%).

When the resulting amplicon was observed to be unique and displaying the expected molecular weight (411 bp), it was sent for sequencing to the Bank of DNA Service of the University of the Basque Country (SGIker sequencing service of UPV/EHU), using both the forward and reverse primers.

### 2.6. Study of the OvoA Transcription Patterns in Mussel Gonads

#### 2.6.1. RNA Extraction

After histologically assigning sex and gametogenic stage to each sampled mussel, six individuals per sex and gametogenic stage from Plentzia and from Arriluze were chosen in order to carry out qPCR analyses. In the Cu exposure experiment, six individuals per sex and per experimental group were selected for extraction of RNA from all tissues. 50–100 mg from each tissue was mechanically and individually homogenized in 1 mL of TriZol^®^ Reagent (Invitrogen, Carlsbad, CA, USA) using a Precellys 24 homogenizer system (Bertin Technologies, Montigny-le-Bretonneux, France) with 1.0 zirconia/silica beads (BioSpec, Bartlesville, OK, USA). RNA extraction was performed following TriZol^®^ Reagent manufacturer’s instructions. RNA yield of each sample was quantified by measuring their absorbance at 260/298 nm and 260/230 nm in a Biophotometer (Eppenorf, Hamburg, Germany). Additionally, RNA quality was analyzed by capillary electrophoresis using the Lab-on-a-Chip kit in an Agilent 2100 Bioanalyzer, according to the manufacturer’s instructions and their Agilent RNA 6000 Nano Kits Assay Protocol (Agilent Technologies, Santa Clara, CA, USA).

#### 2.6.2. cDNA Synthesis and Concentration Measurement

First-strand cDNA synthesis was performed using the Affinity Script Multiple Temperature cDNA Synthesis Kit (Agilent Technologies). cDNA was prepared using 1.2 μg of target total RNA in a total volume of 20 μL. Single-stranded cDNA concentrations were quantified using the QuantiT™ OliGreen^®^ Kit (Life Technologies™, Thermo Fisher Scientific, Waltham, MA, USA), following the manufacturer’s instructions. All measurements were done in triplicates in a black 96-well plate (Corning Incorporated, Corning, New York, NY, USA). Fluorescence in each sample was measured in a Synergy HT Multi-Mode Microplate Reader (Biotek, Winoosky, VT, USA) at standard fluorescein excitation and emission wavelengths of 480 nm and 520 nm, respectively. 

#### 2.6.3. qRT-PCR Analysis of OvoA Transcription Levels in Mussel Tissues

qPCR amplifications were performed using FastStart Universal SYBR Green Master Mix (Rox) (Roche Diagnostics, Mannheim, Germany). Specific primes were FW (5′-3′) GTCCATGGTGGGGAC and RV (5′-3′) AACTCGGATCATTGG, which resulted in an amplicon of 179 bp. First, serial dilutions of a pooled mix of cDNA of all the samples was amplified to determine the best cDNA and primer concentrations. cDNA dilution of 1:20 and primer concentration of 6.25 pmol were determined as the best suited based on cycle threshold (Ct) values (20 < 30) and the non-appearance of primer dimers in the dissociation curve. Three replicas of each sample were amplified on 96-well reaction plates using the 7300 Real-Time PCR System thermocycler (Applied Biosystems) in a total volume of 20 μL, with 2 μL of diluted cDNA. The amplification protocol was programmed to follow an initial denaturation and activation step at 50 °C for 2 min and 95 °C for 10 min, followed by 40 cycles of 95 °C for 15 s and a primer annealing temperature of 58 °C. Amplification reaction was followed by a dissociation stage (95 °C for 15 s, 60 °C for 1 min and again 95 °C for 15 s), where a single peak confirmed the specificity of the selected primer set and the absence of primer dimers.

Efficiency of amplification (E_amp_ = 1.522 in the Cu experiment and 1.94 in the gametogenic cycle analysis) was estimated by calculating a standard curve, after amplification of serial dilutions of a cDNA pool and applying the following equation:

Efficiency_amp_ = 10^(−1/slope)^

All gene transcription results were normalized with the amount of cDNA charged in the qPCR according to Rojo-Bartolomé et al. [24] using an adapted ΔCT formula (*RQ*) with efficiency correction (*E*).

In the sex comparison of gametogenic stages, the reference group was the average of indeterminate gonads per sampling site. In the Cu exposure experiment, the reference group was the day 3 control group and, finally, when comparing transcription in different mussel tissues, the average of all values was used as a reference.

### 2.7. Metabolome Analysis

#### 2.7.1. Sample Preparation for Metabolome Analysis

Mantle samples of six mussels in stage 2 (early gametogenesis) and 5 (ripe gonad previous to spawning) were selected in each site and, additionally, individuals at stage 4 (late gametogenesis) from Plentzia were also processed. In the copper experiment, mantle of six individuals in control and exposure groups were selected, additionally including samples of other tissues (foot, gill, and digestive gland). Mussel samples were wet-weighted and placed in polypropylene cryotubes with 1.4 zirconium oxide beads (Precellys, Bertin Technologies). Prior to the extraction, the samples were spiked with deuterated L-Methionine to provide a final concentration in vial of 5 ng µL^−1^ as an internal standard for quantification. Samples were homogenized in a Cryolys cooling system coupled to a Precellys 24 refrigerated homogenizer system (Bertin Technologies) at 4 °C.

The extraction consisted in the addition of solvents and homogenization in three steps: (i) 400 µL of MeOH and 100 µL of distilled water were added and homogenized during 2 cycles of 50 s at 6400 rpm, (ii) 100 µL of MeOH, 100 µL of distilled water and 200 µL of chloroform were added and vortexed, following the same homogenization cycles and finally, (iii) 300 µL of chloroform and 200 µL of distilled water were added and vortexed, followed by the homogenization cycles again. Then, samples were centrifuged at 14,000 rpm for 15 min. The upper polar phase was recovered on chromatography vials. In order to assure the highest recovery of the polar compounds, a second wash of the non-polar phase was performed by adding to the remaining chloroform the same polar phase as done previously (500 µL of MeOH and 400 µL of distilled water), in one single step. It was vortexed and homogenized likewise, centrifuged, and the upper polar phase was pooled with the first one and kept frozen until analytical analysis.

#### 2.7.2. Analytical Method

Samples were analyzed by liquid chromatography tandem high-resolution mass spectrometry using a Thermo Scientific Dionex UltiMate 3000 Ultra High Performance Liquid Chromatography (UHPLC) coupled to a Thermo Scientific Q Exactive quadrupole-Orbitrap mass spectrometer (LC-qOrbitrap MS), equipped with a heated electrospray ionization source (HESI, Thermo Scientific, Palo Alto, CA, USA). Samples were processed in two different analysis runs so the results could be better compared among experimental groups, the first one including all analyzed mussel mantle samples and the second one including different mussel tissues and *P. lividius* ovaries as controls for the targeted analysis and quantification of OSH levels. Analytes were separated onto a Hydrophilic Interaction Chromatography (HILIC) column Acquity UPLC Ethylene Bridged Hybrid (BEH) Amide (1.7 µm, 2.1 × 100 mm, provided by Waters). Mobile phases used were (A) water as aqueous phase (5 mM ammonium formate and 0.1% formic acid) and (B) acetonitrile as organic phase: (5 mM ammonium formate and 0.1% formic acid). All solvents were of liquid chromatography technical grade (PanReac AppliChem, Castellar del Vallès, Spain). The flow gradient consisted in 5 min of constant B flux at 3%, followed by a constant increase rate until 95% B after 5 min, kept constant for 2 more minutes. Initial conditions were recovered after 4 min.

Molecular and fragment ions were identified with high-resolution Full Scan confirmation mode (FullMS-ddMS^2^) on positive polarity. Resolution was kept at 70,000 in MS and 17,500 for the dd-MS^2^. Scan range went from m/z 70 to 1000 with an automatic gain control (AGC) target of 1e^6^ and an auto maximum injection time (IT) in the Full MS. Fragmentation in the MS2 was achieved with a normalized stepped collision energy ((N) CE) of 10, 35 and 75 eV, with a first mass fixed at 50 m/z and an Automatic Gained Control (AGC) target of 4 e^4^ and an auto maximum IT, with a tolerance of 5 ppm.

#### 2.7.3. Targeted Metabolite Analysis

The confirmation list included 5 target compounds and the internal standard, as well as the 3 target OSHs and their oxidized forms (Supplementary Table S1). Standards were purchased from Sigma-Aldrich (Merck, Darmstadt, Germany), dissolved in H_2_O:methanol (75:25) and stock solutions were kept at −20 ˚C. Working solutions were freshly prepared in methanol.

Quantification was performed following the internal standard method using the spiked labeled methionine and a standards calibration curve between 0.5 and 50 ppm using Xcalibur software (Thermo Scientific). As we did not have an OSH standard, all OSH forms were quantified using an ergothioneine standard calibration curve. Method recoveries of the target analytes were checked with 10 replicates of spiked water following the same procedure as the samples included within the same batches, providing recoveries ranging from 98% for cysteine and 166% for reduced glutathione. Results are provided in µg per mg of wet material. Table S1 in the supplementary information includes full details on chemical characterization.

### 2.8. Statistical Analysis and Data Analysis

All statistical analyses for transcription level data were performed using the Statistical Package for the Social Sciences (SPSS 24.0 for Windows, IBM Spain, Santa Hortensia, Spain). All data were tested to analyze whether they followed a normal distribution. As data did not show a normal distribution, non-parametric analyses such as the Kolmogorov–Smirnoff test was employed coupled with Dunn’s test. Significance was established at *p* < 0.05. The Pearson correlation test was applied to determine the correlation between OSH-A and GSH levels in the mantle samples from Arriluze and Plentzia (*p* < 0.05).

The LC-qOrbitrap data was treated by Compound Discoverer 2.1 (Thermo Fisher Scientific) for the non-target mode of analysis. Briefly, following a standard untargeted metabolomic workflow with statistical analysis, the program performed a set of tasks such as retention time alignment, detection of compounds, predict compositions and molecular formula assignment, among others, to identify compounds (features) present. Finally, the features were compared with internal MS^n^ libraries (mzCloud) and external ones (polar endogenous metabolites from Mass Bank, ChemSpider, etc.), as well as external mass lists, resulting in a list of potential candidates to fulfil features identification, including matching scores for the candidates. Concurrently, basic and advanced statistical treatments (trend analysis, data filtering, principal component analysis, etc.) were used to select the most significant compounds based on the scoring results and the discrimination capacity among the biological groups (Arriluze versus Plentzia, sex or maturity stage). Based on this selection, the data was treated with the web application Metaboanalyst 4.0 (http://www.metaboanalyst.ca) to explore further statistical procedures (e.g., analysis of variance (ANOVA) or partial least squares-discriminant analysis (PLS-DA)) and pathway enrichment analysis. The latter analysis was also carried out with the Mbrole 2.0 web application (http://csbg.cnb.csic.es/mbrole2/). 

## 3. Results

### 3.1. OvoA Gene Sequence Identification in the Mussel (Mytilus Galloprovincialis)

**Figure 1:**
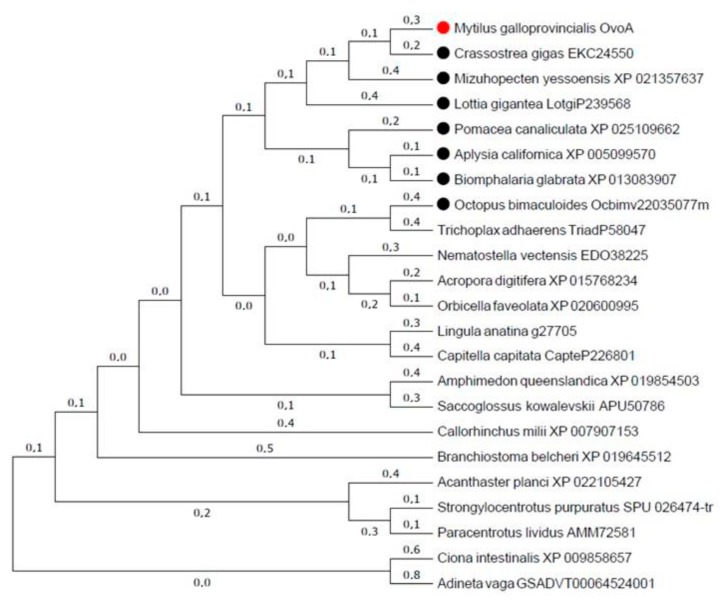
Phylogenetic tree showing the relationship between different animal-deduced ovothiol synthase protein sequences. Name of species is shown together with protein accession numbers either in Genbank or in Ensembl. All mollusc sequences are labelled with a dot. The evolutionary history was inferred by using the Maximum Likelihood method based on the JTT matrix-based model. The tree with the highest log likelihood (−11084, 7135) is shown. Initial tree(s) for the heuristic search were obtained automatically by applying Neighbor-Join and BioNJ algorithms to a matrix of pairwise distances estimated using a JTT model, and then selecting the topology with superior log likelihood value. The analysis involved 23 amino acid sequences. All positions containing gaps and missing data were eliminated. There was a total of 312 positions in the final dataset. Evolutionary analyses were conducted in MEGA7.

Partial coding domain sequence (CDS) of the *ovoA* gene was amplified and sequenced using degenerate primers. The length of the sequenced amplicon was 388 bp long, covering 16% of the *ovoA* coding domain sequence of the *S. purpuratus* orthologous gene. This sequence has been published in the NCBI GeneBank Nucleotide database with accession code MH260573. The obtained sequence was used to query the Expression Sequence Tag (EST) and Sequence Read Archive (SRA) databases through BLASTn, obtaining information to in silico assemble the whole coding domain sequence of mussel *ovoA* (sequence published in the NCBI Third Party Annotation (TPA) section with access code BK012004). The protein deduced from this sequence (Supplementary Figure S1) is formed by 793 amino acids and shows more than 69% amino acid identity with *Mizzuopecten yessoensis* ergothioneine biosynthesis protein 1-like isoform X1 (or OvoA), and 65.35% identity with *P. lividus* OvoA. Phylogenetic analysis clearly clusters mussel OvoA with ortholog sequences of other bivalve molluscs (Figure 1 and Supplementary Figure S1). The protein shows a conserved tripartite domain structure with a very remarkably conserved DNA damage-inducible (DinB) domain, and more divergent FGE sulfatase and SAM methyltransferase domains (Supplementary Figure S1). The putative residues necessary for binding of cysteine and histidine are identical to the ones displayed by sea urchin OvoAs. Instead, residues belonging to the SAM-binding site are less conserved (Supplementary Figure S1).

### 3.2. OvoA Transcription Levels

**Figure 2:**
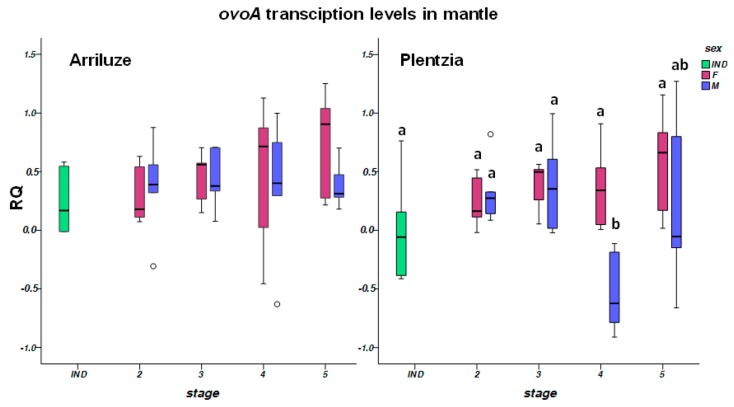
Relative transcription levels of *ovoA* in the mantle of mussels from Arriluze and Plentzia in different gametogenic stages. Boxes in dark and light green correspond to mussels with undifferentiated gonads. Box plots represent the data within the 25th and 75th percentiles, with the median indicated by a line, and top and bottom whiskers indicating the minimum and maximum values (*n* = 6 individuals per experimental group). No significant differences were observed between means in Arriluze. Different letters denote significant differences between means in Plentzia (*p* < 0.05).

**Figure 3:**
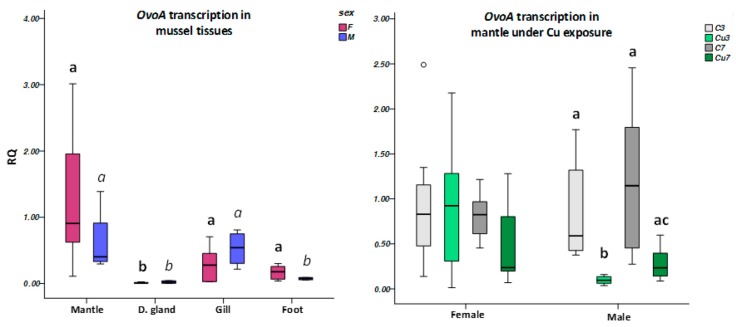
Relative transcription levels of *ovo-A* in the mantle of mussels exposed to copper and in different mussel tissues. Box plots represent the data within the 25th and 75th percentiles, with the median indicated by a line, and top and bottom whiskers indicating the minimum and maximum values (*n* = 6 individuals per experimental group). The first graph shows transcription levels in male and female mantel after 3 and 7 days of exposure to copper respect to controls. The second graph represents the transcription levels in four different tissues of male and female mussels collected in Plentzia in May. Where significant differences were detected, they are indicated by different letters on top of the Box plots (*p* < 0.05). In the tissue comparison, bold letters are used to indicate differences between female tissues and letters in italics to indicate differences in males. No significant differences were observed in female mantle.

*OvoA* was transcribed both in male and female mantle, but also in foot muscle, gills and digestive gland of mussels from both Plentzia and Arriluze (Figure 2 and Figure 3). Transcription levels were low in digestive gland and foot. Similar transcription levels were measured in mussels from both sampling localities (Arriluze and Plentzia), and in mantle of both males and females (Figure 2). Along gametogenesis, females showed a trend towards upregulation through oogenesis in Arriluze (Figure 2), but no significant differences (*p* > 0.05) were observed between mantles with ovaries in different gametogenic stages.

Mussels utilized in the Cu exposure experiment and for the comparison of tissue level transcription were at stage 4 of oogenesis and spermatogenesis. Cu produced a significant downregulation of *ovoA* in male mantles at day 3 of exposure (Figure 3). Instead, transcription was significantly upregulated at day 3 in the digestive gland of female mussels exposed to Cu, although at day 7, transcription levels were higher than at day 3 in all digestive glands, both in control and in Cu-exposed females (Supplementary Figure S2). This trend to significant upregulation with experimentation time was also observed in the gills of males and females (Supplementary Figure S2).

### 3.3. Non-Targeted Metabolome Analysis

**Figure 4:**
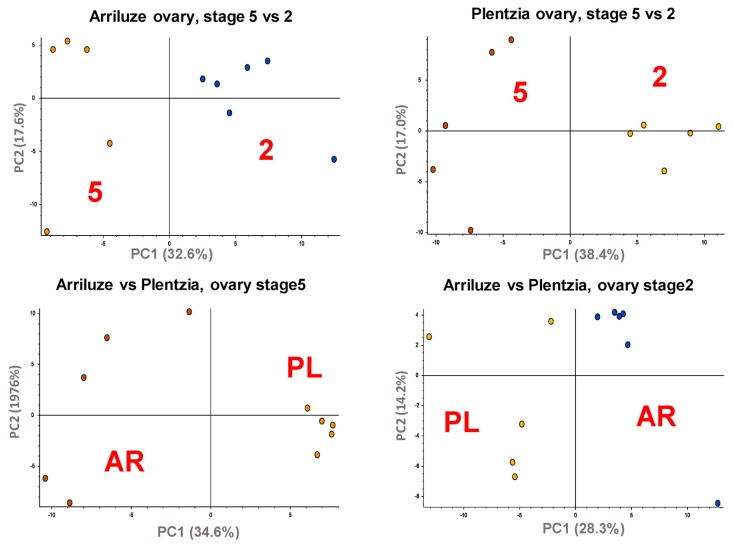
Principal component analysis (PCA) for pairwise comparison of metabolomic profile differences in the mantles of sample groups. PCAs cluster mantle samples according to the site or sampling (PL versus AR), and maturity stage within each sex (2 versus 5).

To obtain a general view of the metabolomic differences between mantle tissues (female versus male), gametogenic stages (2 versus 5, and 4 in the case of Plentzia females), and site of collection (Arriluze versus Plentzia), the data were subjected to principal component analysis (PCA). As shown in Figure 4, the first component clearly establishes differences between developmental stages within one sampling locality and among sampling localities within each developmental stage.

**Figure 5:**
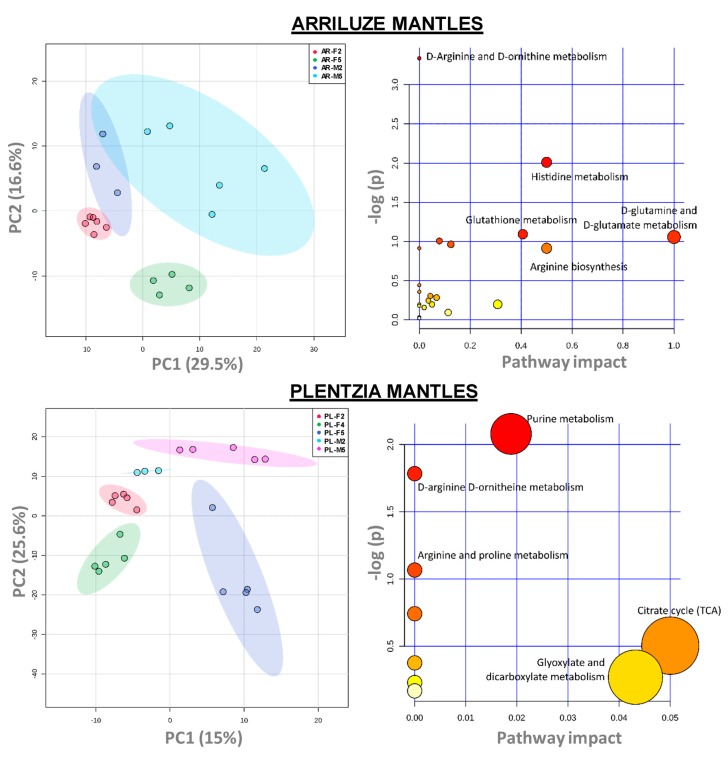
Metabolic pathway analysis comparing male and female mantle from Arriluze. Score projection (PC1-PC2) of the partial least squares-discriminant analysis (PLS-DA) analysis of the metabolites of male and female mantles in Arriluze and Plentzia show clustering of samples according to sex and gametogenic stage. Pathway analysis plot (impact pathway) of the most significant metabolites identified in the previous analysis in Arriluze and Plentzia is also shown. The color and size of each circle are based on *p*-value and pathway impact value, respectively. The most impacted pathways having high statistical significance scores are indicated. *Caenorhabditis elegans* pathway library was used for the analysis. Supplementary Tables S2 and S3 show Mbrole 2.0 results indicating levels of significance and amounts of metabolites.

The PLS-DA analysis of all mantles per sampling site was carried out in order to highlight the most impacted metabolic pathways with gametogenesis. As shown in Figure 5, the first two PCs explain above 45% of the total variance in both cases and the PC1-PC2 score plots show a clustering of the samples according to their sex and gametogenic stage in both sites.

Taking into account the most significant metabolites of each independent analysis (i.e., those with *p* < 0.1 in the ANOVA and VIP–variable importance in projection-level above 1.5 in the PLS-DA), we were able to select between 70 and 100 endogenous metabolites that were used to perform the pathway and enrichment analysis. In the case of Arriluze, the metabolism of D-arginine and D-ornithine appeared as the most significantly impacted pathway (Figure 5, impact pathway), with histidine metabolism, cysteine and methionine metabolism and glutathione metabolism among the most impacted ones (Supplementary Table S2). On the contrary, in the case of Plentzia, the most impacted pathway besides D-arginine and D-ornithine metabolism is the purine metabolism (Figure 5 and Supplementary Table S3).

Comparison of all analyzed female mantles from Plentzia and Arriluze in the PCA score plots clustered metabolite data into distinct groups, and only two pathways appeared repeated to any of the pathways in the previous comparison: histidine and glutathione metabolism (Supplementary Table S4).

### 3.4. Targeted Metabolite Analysis: Ovothiol (OSH) and Glutathione (GSH)

**Figure 6:**
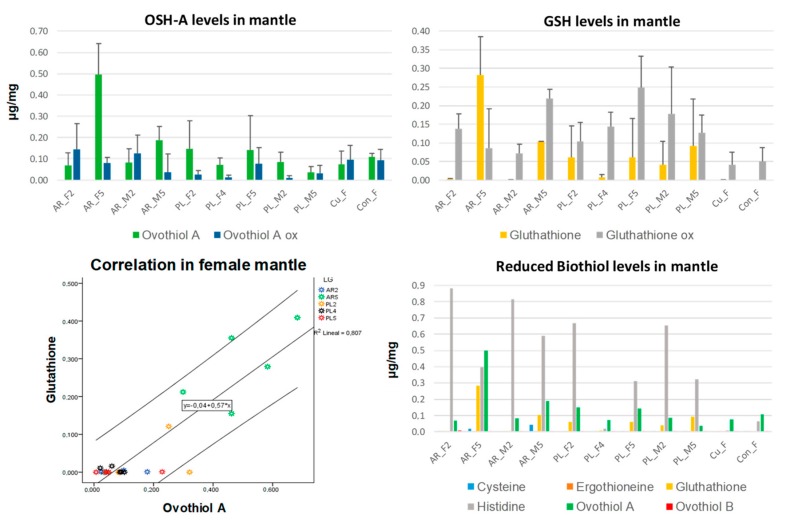
Ovothiol A and glutathion concentrations in mantle of male and female mussels from Arrilizue and Plentzia. Graphs show the concentration of reduced and oxidized biothiols in the mantles (F = female; M = male) of mussels from Arriluze (AR) or Plentzia (Pl) at different stages of gametogenesis (2, 4 or 5). Concentrations in female mantle of mussels exposed to copper (Cu) for 7 days is also shown together with that of their respective controls. Correlation analysis between mantle concentration in reduced OSH-A and GSH is also shown.

**Figure 7:**
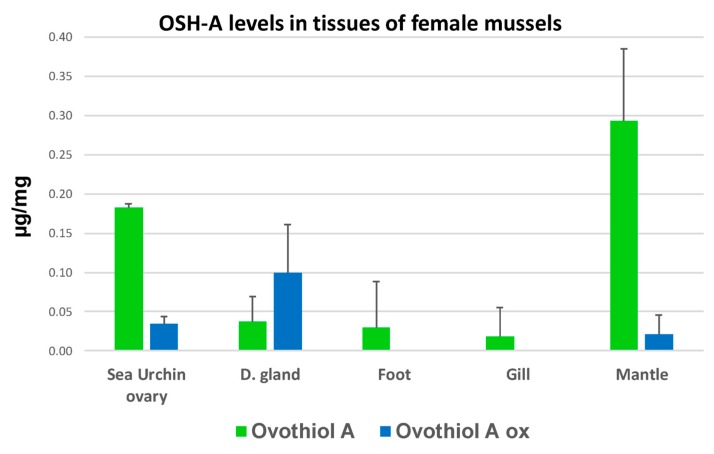
Ovothiol A concentrations in tissues of female mussels. Graph shows the concentrations of reduced and oxidized OSH-A in tissues of mussels captured in Plentzia at stage 4 of oogenesis. Concentrations in mature *P. lividus* ovary are also shown for comparative purposes.

Targeted analysis allowed identification and quantification of OSH-A and OSH-B in their reduced and oxidized forms in all tissues analyzed: female and male mantle, gill, digestive gland and muscle (Figure 6 and Figure 7). Reduced OSH-A is the most prominent form of OSH in mussel tissues, with very little traces of OSH-B (Figure 6). No OSH-C could be distinguished in the chromatograms and only a residual amount of ergothioneine. Levels of OSH-A always exceeded those of GSH (Figure 6). The levels in OSH-A identified in mantles with mature ovaries (Stage 4) of mussels from Plentzia were higher, nearly twice as much, than those identified in mature ovaries of sea urchins *Paracentrotus lividus* analyzed as positive controls (Figure 7).

The levels of OSH-A were significantly increased in the mantles with fully mature ovary, stage 5, of mussels from Arriluze in comparison to stage 2 mussels from the same place (5 times higher levels) or to female mantles from Plentzia at any stage of oogenesis (Figure 6). Male mantle did not show any pattern of alteration in concentrations. This increased concentration of OSH-A in female mantle from Arriluze was positively correlated with the increased levels of GSH, such levels being at their highest also in stage 5 female mantle from Arriluze (Figure 6). The increase in OSH-A levels in female mantle stage 5 in Arriluze occurred together with a reduction in the concentration of histidine (Figure 6). Male mantles also showed an increase in GSH levels towards maturation in Arriluze, but only the female mantles at stage 5 in Arriluze showed higher levels of reduced than oxidised GSH (Figure 6).

## 4. Discussion

In the present work, we showed the coding domain sequence of the first and rate limiting enzyme in the OSH-A biosynthetic pathway, *ovoA*, in the mussel (*Mytilus galloprovincialis*). Its transcription levels were analyzed in mantle along gametogenesis, and in other organs comparing patterns between mussels from two estuaries with different pollutant burdens. The levels of OSH-A and glutathione were quantified following a metabolomic analysis of the hydrophilic metabolites present in the studied tissues and found to increase in female mantle in advanced gametogenesis in the polluted site.

### 4.1. OvoA in M. galloprovincialis and Gene Transcription Levels

*OvoA* whole coding domain sequence was obtained from *Mytilus galloprovincialis.* This was found to be closely related to other orthologous sequences in different molluscs and other marine invertebrates. This was to be expected after the thorough phylogenetic analysis of the evolution of the gene carried out by Gerdol et al. [15], pointing to a monophyletic origin of the gene in metazoa with multiple genes losses in different groups, notably in Ecdysozoa and non-chondrychthyan vertebrates, and acquisition by lateral gene transfer in Bdeloid rotifers and Hydrozoans [15]. As a consequence of the paucity of knowledge on the protein and its physiological function in invertebrates, orthologous genes have received alternative names, such as *ergothioneine biosynthesis protein 1-like*, *5-histidylcysteine sulfoxide synthase*, *meiotically upregulated gene 158 protein-like*, as it can be seen in Castellano et al. [9] or in GenBank. The deduced amino acid sequence shows the common tripartite structure with a well conserved carboxyl terminal DinB superfamily domain containing its typical HX3HXE iron-binding motif, and less conserved FGE-sulfatase and SAM-transferase domains [9,15]. Therefore, the sequence of this gene is quite conserved reflecting the importance of its function.

The tissue-specific patterns of *ovoA* transcription along development or under environmental changing conditions has not been studied and some information only exists for sea-urchins and the starlet sea anemone [9,25]. *ovoA* transcript levels could constitute an indirect method to analyze the pattern of OSH accumulation that has been nearly exclusively studied in oocytes and fluids of some invertebrates and never along development or gametogenesis in gonads [2]. Presently, we found transcription of *ovoA* both in female and male mantle, with similar levels of transcription in both sexes, and likewise in foot muscle, gills and digestive gland. Transcription-levels are highest in mantle. No significant changes in gene transcription were observed along gametogenesis in mantles of both sexes

*OvoA* transcription studied in sea-urchin embryos has been demonstrated to be widespread along development, well before sexual differentiation and ovary formation [9]. Due to its function in ROS scavenging, and its high reactivity with H_2_O_2_ (pKa = 1.4) [2], it is not strange that *ovoA* is expressed and OSH-A is widely produced. We have observed *ovoA* transcription outside female mantle (oocytes) in all tissues tested. We hypothesized that along oogenesis, there would be an increase in *ovoA* transcription from the resting to the spawning phase in order to accumulate OSH-A in mature eggs. Oocytes before spawning are involved mainly in reserve building processes for the latter embryo, and gene transcription rhythms analyzed along oogenesis have demonstrated a peak in transcript abundance at the ripe phase [26]. The same has been reported to occur along spermatogenesis [26]. OSH-A accumulation could be following this increase in transcriptional activity concentration picking just previous to spawning. In the case of spermatogenesis, this could serve as a protective measure along differentiation but specially in oocytes, where it could have additional functions. Broadcast spawners, such as marine bivalves, spawn their gametes in the environment where exposure to environmental variables and pollutants may increase the production of ROS, as it has been proven in oyster oocytes exposed in vitro to dispersed oil [27] or to toxic dinoflagellates [28]. Similarly, fertilization and early embryo cell cleavage is characterized by an increase of H_2_O_2_ production in marine invertebrates [1,4,9] and antioxidants accumulated in oocytes are bound to counteract its pro-oxidant effect. On the contrary, our results showed that *ovoA* transcription levels are constant along oogenesis, and along spermatogenesis. Surprisingly, no studies are available about the dynamics of ROS production and antioxidant gene transcriptional dynamics along bivalve mollusc gametogenesis or early embryo development. 

OSH possesses a thiol group that besides quenching ROS, can also bind metals, and thus play an important role in their detoxification. This is, for instance, illustrated by the cysteine-rich metallothionein proteins (MT10 and MT20) that sequester metals in *M. galloprovincialis* [19] and that are strongly regulated under metal exposure and in polluted environments. This is why *mt20* transcription levels have been proposed as biomarkers of metal exposure in pollution monitoring programs [19]. Metallothionein genes and, at least in sea-urchin genomes, also *ovoA* [9], contain metal-responsive elements, so *ovoA* could respond to metal exposure for the production of more OSH-A. This has been demonstrated in sea-urchin (*P. lividus*) fertilized eggs, and from blastula to pluteus stage larvae under exposure to copper, cadmium and manganese [9]. Arriluze in the mouth of the Bilbao estuary is a polluted site with high concentrations of chemicals and metals, a consequence of industrial and harbor activities, especially in comparison to Plentzia where the presence of metals is very low [29,30]. The results we obtained for mantle *ovoA* transcription levels though, did not show differences between the two sampling sites, neither during oogenesis nor during spermatogenesis. This lack of transcriptional regulation under exposure to chemicals could be perfectly explained by the mixed toxicity in Arriluze, where, together with metals and pro-oxidants, we can find many other chemical compounds, such as organometallics, polycyclic aromatic hydrocarbons, alkylphenols, etc. [29]. We also investigated the transcription patterns of the *ovoA* gene in tissues of adult mussels exposed to Cu, a metal that is known to induce metallothionein gene transcription, to increase ROS production and lipid peroxidation or to alter antioxidant enzyme activities in tissues of mussel species [17,19,31,32]. Our results did not reveal any upregulation of *ovoA* in mantle, but on the contrary, a significant downregulation in male mantle and an upregulation in the digestive gland of female mussels at day 3 of exposure. Such upregulation in the digestive gland occurs in relation to very low transcription levels in controls. In general, upregulation of *ovoA* was observed with time, mussels (control and Cu-exposed) presenting higher transcription levels at day 7 than at day 3, possibly in relation to the laboratory set up conditions. 

The concentration of Cu selected in the present experiment could have been inadequate to obtain a more specific response in the mantle, or maybe the Cu exposure was conducted during a mussel gametogenic stage (Stage 4), at which *ovoA* is non-responsive in mantle. *ovoA* was downregulated in sea urchins after fertilization and then further upregulated in the pluteus stage [9]. In that study, OvoA activity was linked to the cell signaling function of H_2_O_2_ during early development. Nevertheless, a different response to Cd^2+^ exposure was observed throughout development, with upregulation at the blastula stage (when normal transcription is at its lowest) and a strong downregulation at the pluteus stage (when normal transcription is at its highest). In the case of the starlet sea anemone *Nematostella vectensis*, exposure to crude oil produced a significant oxidative stress that resulted in upregulation of an *ergothioneine biosynthesis protein 1-like* transcript [25]. The authors themselves reported such sequence as a possible *ovoA*. These two studies clearly show that *ovoA* is a gene inducible and highly regulated after xenobiotic exposure, at least in those species.

### 4.2. Metabolic Profile of the Gonads of Mussels from Arriluze and Plentzia

The metabolomic profiling revealed thousands of chemical features (>2000) that upon analysis, allowed us to differentiate females from males, mantles from Arriluze from mantles from Plentzia and mantles within different stages of oogenesis or spermatogenesis. Metabolite enrichment analysis and pathway analysis showed the pathways establishing main differences between analyzed “experimental” groups, these including all analyses involving ovaries from Arriluze the histidine metabolism and the glutathione metabolism pathways. In a recent work by Tsentalovich et al. [33], the lens- and gill-specific metabolite profiles in two freshwater fish species highlighted the histidine and glutathione metabolism pathways among the most impacted ones when comparing two different sampling seasons. The only vertebrates to present the *ovoA* gene in their genomes are chondrichthyans [9,15], but it has been proven that OSH-A is accumulated in some fish cells and tissues, including oocytes in salmons [1], and very importantly, the lens in different species [33,34]. Probably, teleosts and other vertebrates accumulate OSH-A through the diet. Quantification of the levels of metabolites (combination of liquid chromatography –LC-, mass spectrometry –MS-, and nuclear magnetic resonance –NMR-) in freshwater fish lenses revealed that OSH-A it is the most important antioxidant present, and that when concentrations of OSH-A increase in autumn in comparison to winter, it occurs together with increased levels of GSH [33]. OSH-A is the result of the conjugation of cysteine and histidine, so that could be one of the drivers of the observed alterations in the histidine and cysteine metabolism pathways in mussel mantles. It is convenient to explain that by not being included in the Compound Discoverer databases, OSH-A is not incorporated in the results shown for our pathway impact analysis, so the impact on the histidine and cysteine metabolism pathways is still higher.

We have demonstrated the presence OSH-A for the first time in marine mussels and also its monomethylated form OSH-B, both in their reduced and oxidized forms in all tested tissues, including ovary and testis. OSH-C was not distinguishable. OSH-A has been described in eggs and biological fluids of many marine invertebrates [1,2,6,9]. OSH-B has been previously described in the ovaries of the scallop *Chlamys hastata*, while OSH-C has only been described in the eggs of the sea urchins *Sphaerechinus granularis* and *Strongylocentrotus purpuratus* [2]. The specific quantification of OSH and GSH levels clearly shows an increased accumulation of the reduced form of OSH-A and of GSH only in the ovaries of mussels from Arriluze. This is not matched by anything similar in testis, or in ovaries from Plentzia nor in gonads from mussels from Plentzia exposed to Cu. This accumulation in stage 5 ovaries from Arriluze is very potent and one of the highest concentrations of OSH-A ever described in any species, exceeding those identified in sea-urchins, as it can be observed through our comparison with *P. lividus* ovarian mantle levels. The concentration measured in Arriluze stage 5 female mantles was 2.5 µmol/mg wet tissue. The concentration measured with the same technique in mature *P. lividus* ovaries was 0.9 µmol/mg wet tissue, similar to the 1.1 µmol/mg tissue measured by Castellano et al. [9] in newly fertilized sea-urchin eggs or embryos. 

As explained above, Arriluze in the opening of the Bilbao estuary, is a contaminated site [30], and there is evidence that exposure to xenobiotics provokes increased ROS production, increased antioxidant enzyme activities and modified GSH and glutathione disulfide (GSSG) levels (altered GSH/GSSG ratios) in oocytes and mantle of different mollusc species [32,35,36]. Our results reveal that the OSH-A accumulation in female mantle in Arriluze is linked to the oogenic stage, pointing towards an accumulation close to the moment of spawning, possibly anticipating the oxidative stress that spawned oocytes will have to face after spawning and fertilization in polluted waters, and this happens in the absence of apparent *ovoA* transcriptional regulation. Post-transcriptional regulatory mechanisms from mRNA processing to enzyme activity and transport regulation must be involved to allow such a sudden accumulation of OSH-A. OSH-A acts as a non-enzymatic glutathione peroxidase system in consuming H_2_O_2_ but there is no OSH reductase to replenish the stock of reduced OSH-A. The GSH/GSSG system has been presented as the system to replenish the redox balance [2,5,9].

## 5. Conclusions

In this work, we have sequenced and identified the whole coding domain sequence of the *ovoA* gene in the mussel *Mytilus galloprovincialis*. *ovoA* is transcribed in female and male mantle of mussels without alterations in its transcription pattern during gametogenesis. The pollution burden, nutrients and chemicals, present in the estuary of Bilbao at Arriluze, do not modify the pattern of transcription of this gene, but OSH-A is accumulated to very high concentrations in the mantle of female mussels at stage 5 of oogenesis, accumulation that occurred concurrently with that of GSH. In the future, the presence of OSH-A could be monitored in the mantle of female mussels to study possible accumulation in oocytes along gametogenesis in pollution biomonitoring campaigns. This study points to the need for further mechanistic studies to understand the mechanism underlying OSH-A accumulation under controlled and systematic laboratory set-ups analyzing the transcriptional or post-transcriptional effects on *ovoA* of different pollutants and pro-oxidant conditions.

## Supplementary Materials

The following are available online at https://www.mdpi.com/2218-273X/10/3/373/s1, Figure S1: Sequence alignment of the mussel OvoA protein sequence, Figure S2: Relative transcription levels of *ovo-A* in mussel tissues, Table S1: Targeted metabolites and analytical variables, Table S2: Mbrole-pathway enrichment analysis: mantles from Arriluze, Table S3: Mbrole-pathway enrichment analysis: mantles from Plentzia, Table S4: Mbrole-pathway enrichment analysis: females mantles from Plentzia and Arriluze.
