# Supplementary material for: Gametogenesis-Related Fluctuations in Ovothiol Levels in the Mantle of Mussels from Different Estuaries: Fighting Oxidative Stress for Spawning in Polluted Waters
Source: Biomolecules. 2020 Feb 28;10(3):373. doi: 10.3390/biom10030373 (PMC7175103; doi:10.3390/biom10030373)
Supplement: Supplementary file 1 [file biomolecules-10-00373-s001.pdf]

SUPPLEMETARY MATERIAL

Gametogenesis related fluctuations in ovothiol levels in the mantle of mussels from different estuaries; fighting oxidative stress for spawning in polluted waters

Oihane Diaz de Cerio [1,] Lander Reina [1,] Valeria Squatrito [1,] Nestor Etxebarria [2,] Belen Gonzalez-Gaya [2,] Ibon Cancio [1,*]

[1] CBET Research Group, Dept. Zoology & Animal Cell Biology; Faculty of Science & Technology (FCT-ZTF) and Research Centre for Experimental Marine Biology & Biotechnology (PiE-UPV/EHU), University of the Basque Country (UPV/EHU), EMBRC-Spain, Areatza Hiribidea 47, 48620 Plentzia, Basque Country

[2] IBEA Research Group, Dept. Analytical Chemistry; Faculty of Science & Technology (FCT-ZTF) and Research Centre for Experimental Marine Biology & Biotechnology (PiE-UPV/EHU, University of the Basque Country (UPV/EHU), EMBRC-Spain, Areatza Hiribidea 47, 48620 Plentzia, Basque Country

* Correspondence: ibon.cancio@ehu.eus, Tel. 00 34 946017670, (I.C.)

Table S1: Targeted metabolites and analytical variables.

Table S2: Mbrole-pathway enrichment analysis: mantles from Arriluze.

Table S3: Mbrole-pathway enrichment analysis: mantles from Plentzia.

Table S4: Mbrole-pathway enrichment analysis: females mantles from Plentzia and Arriluze.

Figure S1: Sequence alignment of the mussel OvoA protein sequence.

Figure S2: Relative transcription levels of ovo-A in mussel tissues.

Supporting Table S1. Targeted metabolites and analytical variables. Detail on m/z used for calculation of metabolites concentrations. Retention time and average recovery percentage are given for each compound.

COMPOUND	m/z	Retention time (min)	Average Recovery (%)
**d_3-methionine**	154,08110	2,17	-
**Cysteine**	122,02703	2,08	97,9
**Histidine**	156,07675	1,07	126,0
**L-glutathione oxidised**	613,15924	1,02	132,7
**L-gluthathione**	308,09108	2,07	166,2
**Ergothioneine**	230,09577	2,28	127,1
**Ovothiol A**	202,06421	2,23	-
**Ovothiol B**	216,07988	2,33	-
**Ovothiol C**	230,08188	2,60	-
**Ovothiol A_oxidised**	401,10544	2,25	-
**Ovothiol B_oxidised**	429,13657	2,33	-
**Ovothiol C_oxidised**	457,98328	N.F.	-

Supporting Table S2. Mbrole-pathway enrichment analysis: mantles from Arriluze. All mantle samples from Arriluze were analysed, male and female included. Only the impacted pathways are shown and those significantly impacted (p<0,05) are shadowed.

Annotation	in set	in backgrnd	p-vaues	FDR corr.	Matching IDs
Arginine and proline metabolism	9	82	2.04E-06	1.35E-04	C00025 C00750 C00763 C01035 C01110 C02647 C04137 C04282 C15699
D-Arginine and D-ornithine metabolism	3	10	3.24E-04	1.07E-02	C00792 C01110 C03943
Purine metabolism	6	92	1.97E-03	3.33E-02	C00020 C00147 C00212 C00242 C00294 C00387
Glutathione metabolism	4	38	2.05E-03	3.33E-02	C00025 C00051 C00750 C16565
Biosynthesis of alkaloids derived from ornithine, lysine and nicotinic acid	5	67	2.64E-03	3.33E-02	C00025 C00253 C00408 C00417 C10865
Histidine metabolism	4	44	3.53E-03	3.33E-02	C00025 C01152 C03680 C05130
Cysteine and methionine metabolism	4	56	8.38E-03	6.91E-02	C00051 C01005 C01234 C08276
Pyrimidine metabolism	4	59	1.01E-02	7.06E-02	C00380 C00475 C00906 C05100
C5-Branched dibasic acid metabolism	3	32	1.07E-02	7.06E-02	C00025 C00417 C01109
Nicotinate and nicotinamide metabolism	3	44	2.52E-02	1.51E-01	C00253 C01004 C01297
Lysine degradation	3	47	2.99E-02	1.64E-01	C00408 C01142 C04020

Supporting Table S3. Mbrole-pathway and enrichment analysis of mantles from Plentzia. All mantle samples from Plentzia were analysed, male and female included. Only the impacted pathways are shown and those significantly impacted (p<0,05) are shadowed.

Annotation	in set	in backgrnd	p-value	FDR corr	Matching IDs
Purine metabolism	5	92	1.01E-03	4.55E-02	C00147 C00212 C00242 C00294 C00387
Arginine and proline metabolism	4	82	4.96E-03	7.44E-02	C00763 C01035 C02647 C04282
Pyrimidine metabolism	3	59	1.35E-02	1.52E-01	C00380 C00906 C05100
Lysine biosynthesis	2	32	3.01E-02	2.71E-01	C12986 C12987
Nicotinate and nicotinamide metabolism	2	44	5.39E-02	3.69E-01	C00253 C01297
Benzoxazinoid biosynthesis	1	9	7.45E-02	3.69E-01	C15772
Cysteine and methionine metabolism	2	56	8.23E-02	3.69E-01	C01234 C08276
D-Arginine and D-ornithine metabolism	1	10	8.24E-02	3.69E-01	C00792

Supporting Table S4. Mbrole-pathway enrichment analysis: female mantles from Arriluze and Plentzia. All females mantles measured were included in the analysis both from Plentzia and from Arriluze. Only the significantly impacted pathways are shown (p<0,05).

Annotation	in set	in backgrnd	p-value	FDR correction	Matching IDs
Arginine and proline metabolism	9	82	1.33E-09	7.85E-08	C00025 C00315 C00431 C00750 C00763 C02647 C04282 C05933 C15699
Metabolic pathways	20	1455	9.40E-05	2.77E-03	C00003 C00025 C00026 C00043 C00135 C00245 C00315 C00408 C00417 C00499 C00633 C00725 C00750 C00763 C00792 C02378 C02647 C03680 C05100 C15699
Histidine metabolism	4	44	1.73E-04	3.40E-03	C00025 C00026 C00135 C03680
Biosynthesis of alkaloids derived from ornithine, lysine and nicotinic acid	4	67	8.77E-04	1.10E-02	C00025 C00026 C00408 C00417
beta-Alanine metabolism	3	31	1.02E-03	1.10E-02	C00135 C00315 C00750
C5-Branched dibasic acid metabolism	3	32	1.12E-03	1.10E-02	C00025 C00026 C00417
Biosynthesis of alkaloids derived from histidine and purine	3	35	1.46E-03	1.23E-02	C00026 C00135 C00417
Glutathione metabolism	3	38	1.86E-03	1.37E-02	C00025 C00315 C00750
ABC transporters	4	90	2.63E-03	1.56E-02	C00025 C00135 C00245 C00315
D-Glutamine and D-glutamate metabolism	2	13	2.65E-03	1.56E-02	C00025 C00026
Lysine degradation	3	47	3.43E-03	1.84E-02	C00408 C00431 C03656
Reductive carboxylate cycle (CO2 fixation)	2		4.75E-03	2.34E-02	C00026 C00417
Proximal tubule bicarbonate reclamation	2	17	5.36E-03	2.43E-02	C00025 C00026
Pyrimidine metabolism	3	59	6.53E-03	2.75E-02	C02067 C03997 C05100
Citrate cycle (TCA cycle)	2	20	7.40E-03	2.91E-02	C00026 C00417

Supporting Figure S1.

```
OctopusOcbimv22035077m      ---------------------------------MLALRRFRRRRFQLIRS----------T 18
ParacentrotusAMM72581       ------------------------------------------------------------ 0
MytilusOvoA                 ------------------------------------------------------------ 0
CrassostreaEKC24550         MDKAWSCLAAYKPPSQDDDLPSVQGCCNDSESSVVLELFPGSVCVFLLSELCGLTAMIQL 60

OctopusOcbimv22035077m      EQPAREINVQVAEHSTDTCILNVVLGDIQRDTECDKADPLNYRYNTNRKDIQEYFLNSYN 78
ParacentrotusAMM72581       -------------MATELSLEDILTGPYT----WKSMTPC-DLSNCTKQQILDYFENSYS 42
MytilusOvoA                 ------------------MKTSVLTGKEN----FQSLKPP-DLSCCTKQQLQDYFENSYE 37
CrassostreaEKC24550         LCPETYL-AALAKTQGQKMQSKILTGNLT----FKSLRPP-DLSNCSKGELCEYFENSYD 114
                                                 .:: *        ..  *       .: :: :** ***.

OctopusOcbimv22035077m      LYESLFTSIADDSALYLYPDRLRLPLIFYYGHTSAVYYVNKLMLADLLTKRINAEFETIFE 138
ParacentrotusAMM72581       LNESIFAALKTEDAIYKAPDRLRLPLIFYYAHTAVVYINKLMLAGLIEERVNFEYETMFE 102
MytilusOvoA                 LNESLFLSLKEDDIFYKCPDRLRLPLIFYFCHTSVVYVNKLVLSGLLKERVNLEFETMFE 97
CrassostreaEKC24550         LNESLFTSLNDDSVFYKCPDRLRLPLIFYFCHTAVVYVNKLMLAGLIKERVNLEFETMFE 174
                            * **:* ::  :. :*  ***********: **:.**:***:*:.*: :*:* *:**:**

OctopusOcbimv22035077m      TGVDEMFWDDTVNQRIDGKPIVWPEVVDVTAYRRQVKNAILQMINDTPLELPITMESKWW 198
ParacentrotusAMM72581       TGVDEMSWDDTENYRMGG-SYQWPSLKDVVQFRLKVRNVIRKVIEDTPLELPVTQESKWW 161
MytilusOvoA                 TGVDEMSWDDTENYRMGG-SYKWPSVNEVMDYRRTVRNIIIKMIQDTPLVLPITKESPWW 156
CrassostreaEKC24550         TGVDEMSWDDTENYRMGG-SYKWPSLSSVVEYRRNVRNLVLKVIHDTPLDLPVTMESPWW 233
                            ****** **** * *:.*    **.: .*  :*  *:* : ::*.**** **:* ** **

OctopusOcbimv22035077m      ALLMGMAHERIHLETSSVLIRQLPVNVVTKPPTWVYAEMKTGKPVGPNSLVALEGREVQL 258
ParacentrotusAMM72581       AMFMGFEHERIHIETSSVLIRQLPIGMVKTPEQWVPGPLTYGESPSKNPLLPVEGTEVTM 221
MytilusOvoA                 GLLMGMEHERIHLETSSVLWIRQLPVDMVTKPDGWVYGPMKYSEPVMSNPMIRVEEREVTY216
CrassostreaEKC24550         AILMGIEHERIHLETSSVLIRQLPVEMVTKPKGWVYAPFKCGGRVEENPLISVETQDVTF 293
                            .::**: *****:***********: :*..*  ** . :. .     * :: :*  :*

OctopusOcbimv22035077m      GKPRNFPSYGWDNEYGESRCLVPDFEATKYLVTNEEFLKFVTDKGYERQELWSDIGWKWR 318
ParacentrotusAMM72581       GKPKDFPSYGWDNEYPQIQTSVPSFEASKYLITNREFMEFVTDKGYENEKLWSKEGWKWR 281
MytilusOvoA                 GKPDDFPSYGWDNEYGEETSRVPAFEASKYLVTNREYLEFVHDEGYSKKNLWTEEGWGWK 276
CrassostreaEKC24550         GKPKDFPSYGWDNEYGEFSTRVPAFEASKFMITNGEYLEFVESGGYEDEKFWTEEGWKWK 353
                            *** :********** :     ** ***:*:::** *:::** . **. :::*:. ** *:

OctopusOcbimv22035077m      CFRSAIHPTFWVCNKGCKSGCGETLSNYTHCQLRSSDE---------------------- 356
ParacentrotusAMM72581       KYRNARHPTFWVCDKGCVSGCGGVLAGYSHCKPDQN------------------------ 317
MytilusOvoA                 LYRHAEHPAFWVCDNGCKGGCGAALSSYSHCHFNTELTTTNGVTNGQSNIVTNGQSNGLT 336
CrassostreaEKC24550         VYRQSKHPVFWVCNHSCKGGCGADLSKYSPCHFPNSGT--NGVING-------------- 397
                             :* : **.****::.* .***  *: *: *:   .

OctopusOcbimv22035077m      ----------------------------------------LNEPTFCYRAMFDVITLPRD 376
ParacentrotusAMM72581       ----------------------------------------QNSSQYRYRSFCEETAIPWD 337
MytilusOvoA                 NGQNNGVTNGQNNGMTNGQSNGVTNGQSNGVTNGHTNGFNRGGTVYKYRAMFDVLDMPLD 396
CrassostreaEKC24550         -------------SLTNGHSR--------EVING------GEKRTYMYRAMFDVLPLPAD 430
                                                                     : **:: :   :* *

OctopusOcbimv22035077m      WPVEVNYHEAKAYCTWMGPGYRLPTEAEAMLMKGTMPPITERVKCDIIHSGKP--DANIN 434
ParacentrotusAMM72581       WPVEVNYHEAKAYAAWKGPGYRLPTESEHNAMRDKK-PISKGTDSDLIYDKELHQKSNIN 396
MytilusOvoA                 WPVEVNYHEAHAFCSWKGSGNRLLTEAEMNVIRDDQFPPSDGPKSDVIYQDNI--DLNLN 454
CrassostreaEKC24550         WPAEVNYHQARAFCAWKGFGFRLLTEAERNVIKGKKLETTQGIDGDIIYQKKP--KANIN 488
                            **.*****:*:*:.:* * * ** **:*   ::.      :.  . *:*:. :   . *:*

OctopusOcbimv22035077m      FKYGSP------------------------------------------------------ 440
ParacentrotusAMM72581       LTFGTSTPVHMYPANGKGFHDVFGNVWQWVEDHFNGLPGSETHYLYDDFSSPTYDGKHNV 456
MytilusOvoA                 LKFGSSTPVNMFPATEAGFHDVYGNVWHWTEDHFNGLYDYRSHYLYDDFSSPCFDGRHNI 514
CrassostreaEKC24550         LHYGSSTPVNMYPANELGYHDVFGNAWEWAEDHFNGLNGYSSHWYYDDFSSPCFDGRHNL 548
                            : :*:

OctopusOcbimv22035077m      -SNGSWISTGNEASRFARYAFRRHFFQHLGFRMVKSSH-----DIPVKLIGPTDQVTKTS 494
ParacentrotusAMM72581       ILGGSWISTGDEASRFARYAFRRHFFQHAGFRLARSCSDQ---DVPVRLVRNPAHAD--- 510
MytilusOvoA                 ILGGSWISTGDEASRFARYAFRRHFIQHAGFRLARSLETA---ELPARLIDSEVFVLGIG 571
CrassostreaEKC24550         ILGGSWVSTGDEASKFARFAFRRHFIQHAGFRIARSLQLADKINVPARMVKDEVFVLGVG 608
                              .***:***:***:***:******:** ***:.:*       ::*.:::     .

OctopusOcbimv22035077m      ELENGSPVKS---YSQVNVSANSQYRYSDKLVIEDVLLQEYGFKN-SLDAVALQKGLSLM 550
ParacentrotusAMM72581       -QDVSLPLK--DISKKTFMTTNTQLLMETQSCVDERLFDEYVLDDINLSSALGKVSMDIA 567
MytilusOvoA                 VQENPYVIEEEGHKVMKVPTTNKQYGYDEKHQLYGILEQEFGYRD-PLPLAIARLCKEY- 629
CrassostreaEKC24550         VEEKEIPD-ADEVEYKWVPTTNYHYSYDTEDSLYGIIELEFGFRD-TFPHNVAQHCLSLQ 666
                              :                ::* :   . :  :   :  *:   :  :      :   .

OctopusOcbimv22035077m      EEWSIPAKTALVLGSSSGRVAFDMSKTFKKVLGIDYSNRLLSVAEKIKSSKSTIIP---- 606
ParacentrotusAMM72581       RRHGTKVSHALDVGCGCGRMSFELSRAVDQVIGLEYCEVFLKSAEELLSKKQKVFYCPKE 627
MytilusOvoA                 -KTESGNESLCWIGCGTGIGPMHLHNVFQNILAIDYGGRFIDTALKLQKGEQVE------ 682
CrassostreaEKC24550         KKYQCGSKSALWLGSGAGRGPLELTNVFQNVLGVDYAARFIDAAQKLKNGKALS------ 720
                             .     .    :*.. *   :.: ....:::.::*   ::. * :: . :

OctopusOcbimv22035077m      ----------YSLGK----EVEIDPSWSRENVIFKQLTWLSMELGSHNMVLVTFLERLQN 652
ParacentrotusAMM72581       DCRIPAKRVKRSTHDGCTITAKLPTDTQPSRVTLKQFTWIPNELSGFDLVVHHCLDRVPN 687
MytilusOvoA                 ----------YTKSNGQQAVAMFDG-VGYDNVVFKQLTWLPNEVNSHSMTVVTFLDRTLN 731
CrassostreaEKC24550         ----------YKAGNGEQKTAVMSGDYQPDRAIFKQLTWIPNEICDHDMTIVTFMDRTMN 770
                                          .  .     . :       ... :**:**:  *: ...:.:   ::*  *

OctopusOcbimv22035077m      TKAWLYRLWELLKDDGLLVITSKDTTWTAAELQQYLQPR--------------------- 691
ParacentrotusAMM72581       KRAWLIRLWEILMPNGIVVI-SGLGDWTKEKLEPIIGDKLRCMETIPVKYAEDYWCTEPD 746
MytilusOvoA                 PKAWLARLWEVTKEGGLVLIVS---DWESEKLQVSLQRHMVYIGKRELSYQD--GNAVAM 786
CrassostreaEKC24550         AKAWLARLWEVTVKSGLVVIVSETKEWNKDNLAQYFGNNLLFKEAQDLKYTSKKGEETLQ 830
                             :*** ****:   .*:::* *     *    :*    :   .

OctopusOcbimv22035077m      -----------------      691
ParacentrotusAMM72581       KETWATVWCLKTRAEAQ      763
MytilusOvoA                 MTIWKRI----------      793
CrassostreaEKC24550         ITAWARK----------      837
```

Supporting Figure S1. Sequence alignment of the mussel OvoA protein sequence. Alignmnet of mussel OvoA sequence with that of sea-urchin *P. Lividus,* and molluscs *Octopus bimaculatus* and *Crassostrea gigas*. DinB superfamily domain in the N-terminal region is boxed in blue. The putative iron binding motif (HX3HXE) is indicated in red. The FGE-sulfatase domain is boxed in pink and the SAM-transferase domain is boxed in green. The residues belonging to the SAM-binding site are indicated in blue. The putative residues accounting for binding to cysteine and histidine are highlighted in yellow. In bold the deduced mussel protein fragment amplified and sequenced using degenerate primers.

Supporting Figure S2

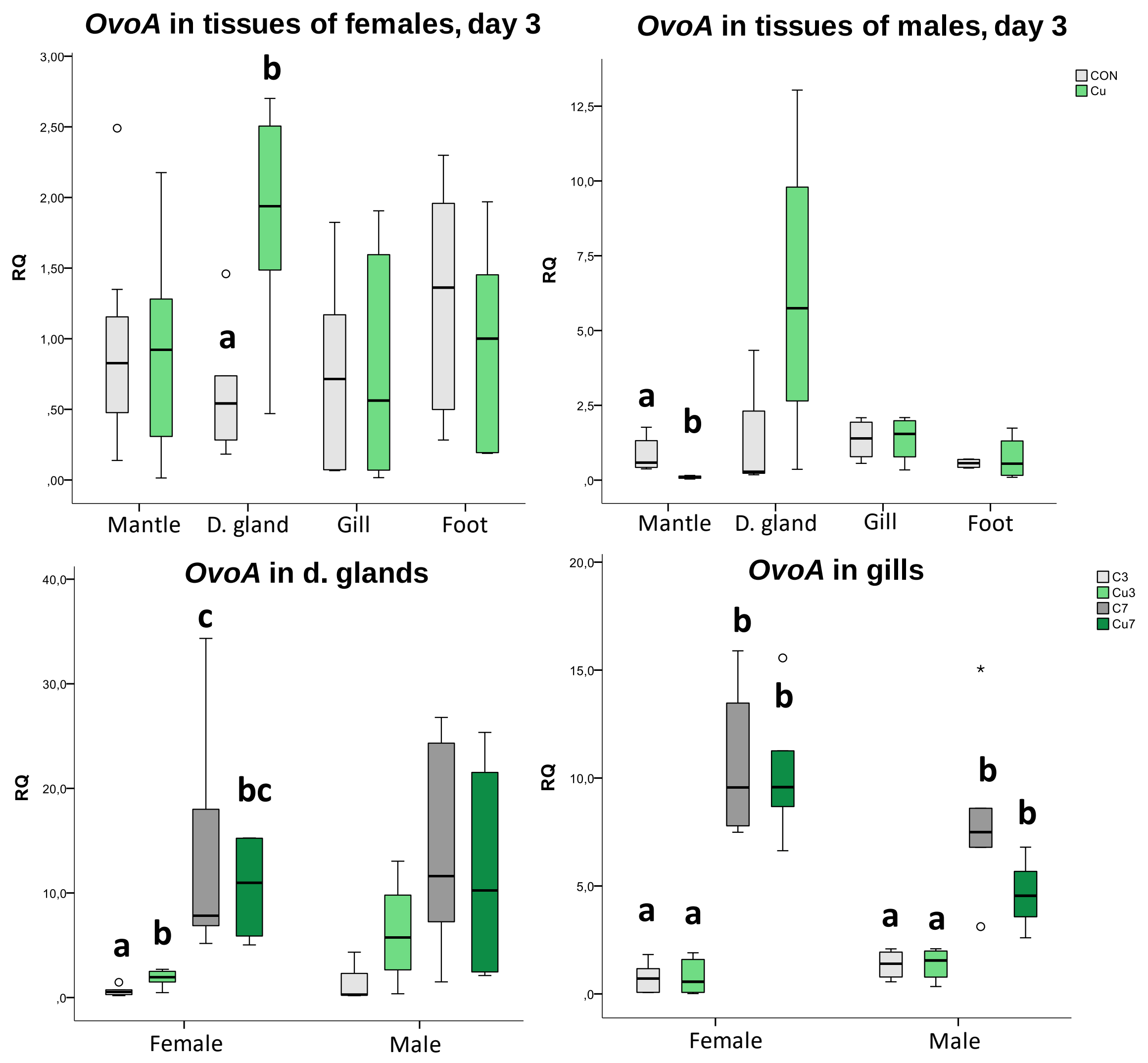

Supporting Figure S2. Relative transcription levels of *ovo-A* in mussel tissues. First two graphs compare the *ovoA* transcription levels across mussel tissues (females and males) at day 3 of copper exposure. Last two graphs show transcription levels in digestive glands and gills of mussels of both sexes exposed to copper for 3 and 7 days. Box plots represent the data within the 25th and 75th percentiles, with the median indicated by a line, and top and bottom whiskers indicating the minimum and maximum values (n=6 individuals per experimental group. Different letters on box plots indicate statistical differences between means ($p<0,05$), in first two graphs within each tissue and in last two within each sex.
